# Association Between Vitamin D Deficiency and Testosterone Levels in Adult Males: A Systematic Review

**DOI:** 10.7759/cureus.45856

**Published:** 2023-09-24

**Authors:** Neetha R Monson, Nimra Klair, Utkarsh Patel, Ayushi Saxena, Dhara Patel, Ismat E Ayesha, Tuheen Sankar Nath

**Affiliations:** 1 Internal Medicine, California Institute of Behavioral Neurosciences & Psychology, Fairfield, USA

**Keywords:** vitamin d and sex steroids, vitamin d deficiency and testosterone, vitamin d and hypogonadism, vitamin d and androgens, vitamin d and testosterone

## Abstract

Vitamin D deficiency and its potential impact on testosterone levels have been subjects of scientific interest. This systematic review aims to evaluate the association between vitamin D deficiency and testosterone levels in adult males and examine the effects of vitamin D supplementation on testosterone. A comprehensive literature search was conducted in accordance with the PRISMA guidelines across PubMed, Google Scholar, and ScienceDirect. The inclusion criteria involved studies published in English between 2013 and 2023, which investigated the correlation between vitamin D and testosterone levels in adult males. The process of data extraction and synthesis encompassed various aspects, including study characteristics, participant demographics, measurement methods, and outcomes pertaining to the association. The initial search resulted in a pool of 354,235 articles. Through the application of relevant filters and the review of titles and abstracts, 48 articles were chosen for further assessment. Out of these, eight studies fulfilled the inclusion criteria and were ultimately incorporated into the final review. The included studies consisted of four cross-sectional studies, three randomized controlled trials (RCTs), and one analysis utilizing Mendelian randomization. The results showed heterogeneity among the studies, as some reported a positive correlation between vitamin D levels and testosterone, while others did not find a significant association. The effects of vitamin D supplementation on testosterone levels were inconclusive, with limited evidence of significant changes in total testosterone. However, potential influences on sex hormone-binding globulin and free testosterone levels were observed. To establish more definitive evidence regarding the impact of vitamin D on testosterone levels, there is a need for further well-designed, long-term RCTs that encompass diverse populations.

## Introduction and background

Vitamin D plays a vital role in maintaining calcium balance and promoting bone mineralization [1,2]. The prevalence of insufficient vitamin D levels across diverse populations and the potential association between inadequate vitamin D status and adverse health outcomes underscore the importance of addressing vitamin D deficiency as a significant public health issue [1-4]. Beyond its classical role in calcium homeostasis, emerging research indicates that vitamin D may have wider physiological implications, extending its impact on multiple organ systems and metabolic pathways [5,6]. Insufficient vitamin D levels are associated with a spectrum of adverse outcomes, including increased risks of osteoporosis, autoimmune diseases, cardiovascular disorders, and certain cancers [3]. These health consequences underscore the importance of addressing vitamin D deficiency as a pressing public health issue that impacts both individual well-being and healthcare systems on a global scale. Of particular interest is the potential interaction between vitamin D and testosterone, a vital hormone in adult males.

The presence of vitamin D receptor (VDR) which is a type of transcription factor belonging to the same nuclear receptor family as testosterone [4,7,8] and vitamin D metabolizing enzymes in the human testis, ejaculatory tract, and mature spermatozoa has piqued scientific interest in understanding the potential impact of vitamin D on male reproductive function [9,10]. Studies have shown correlations between the expression levels of VDRs, and enzymes involved in vitamin D metabolism in spermatozoa, suggesting a possible role for vitamin D signaling in male fertility. Furthermore, VDR and the enzyme responsible for converting vitamin D into its active form, known as CYP27B1 (alpha-1 hydroxylase), have been detected in various cells of the reproductive system [11-13]. These findings suggest that vitamin D may potentially influence fertility, prompting further investigation into its wider physiological consequences.

The shared membership of VDR and testosterone in the nuclear receptor family underscores the significance of understanding their interaction. Therefore, a systematic review is imperative to thoroughly assess the available evidence and present a comprehensive evaluation of this relationship in adult males.

The primary objective of this systematic review is to critically analyze the chosen articles and provide a clear understanding of the current knowledge regarding the association between testosterone and vitamin D levels. Furthermore, it aims to evaluate the potential impact of vitamin D supplementation on testosterone levels.

## Review

Methods

In this study, we adhered to the rigorous and transparent guidelines set forth by the Preferred Reporting Items for Systematic Reviews and Meta-Analyses (PRISMA) [14] to ensure the integrity of our review. To determine the studies to be included, we established explicit inclusion and exclusion criteria, and extensive discussions were held among all authors to reach a consensus. A comprehensive literature search was then conducted using a variety of databases and keywords, which were also collectively agreed upon by all authors.

Inclusion and Exclusion Criteria

Inclusion: Adult males over 18 years of age, topics related to vitamin D deficiency and testosterone levels, research articles published from 2013 to 2023, and articles published only in the English language.

Exclusion: Pediatric population, reviews, case reports, case series, only abstracts, letter to the editor, and commentary; animal studies.

Search Strategy

We performed a systematic search of relevant literature using three prominent databases: PubMed, Google Scholar, and ScienceDirect. In PubMed, we employed Medical Subject Headings (MeSH) keywords, while advanced search strategies were employed in both Google Scholar and ScienceDirect [15-18]. The search yielded a substantial number of pertinent articles that are relevant to the topic at hand.

The following search terms were employed in each respective database: 1) PubMed (using Medical Subject Headings - MeSH): Vitamin D deficiency OR Vitamin D insufficiency OR ( "Vitamin D Deficiency/etiology"[Majr] OR "Vitamin D Deficiency/metabolism"[Majr] ) AND Testosterone OR Hypogonadism OR Androgens OR ( "Testosterone/deficiency"[Majr] OR "Testosterone/metabolism"[Majr] OR "Testosterone/physiology"[Majr] ); 2) Google Scholar (using advanced search strategies): Vitamin D, Vitamin D deficiency, Testosterone, Adult Males; 3) ScienceDirect (using advanced search strategies): Vitamin D, Vitamin D deficiency, Testosterone, Adult Males.

Results

Data Extraction

Initially, a total of 354,235 articles were retrieved from the three designated databases: PubMed, Google Scholar, and ScienceDirect, using the search strategies we aligned on. The individual contributions of these databases were as follows: 177,427 articles from PubMed, 154,000 articles from Google Scholar, and 22,808 articles from ScienceDirect. Following the application of filters and the removal of duplicates, the number of articles was reduced to 2,631 for further assessment. The screening process involved a review of the titles and abstracts of these articles, resulting in a selection of 48 articles. Subsequently, a meticulous evaluation of these 48 articles was conducted, taking into account inclusion and exclusion criteria as well as the availability of full-text articles. This thorough evaluation led to a final set of 18 articles, which underwent a comprehensive quality check. The selection of these articles was achieved through consensus between the two authors NRM and NK involved in the study.

Quality Assessment

Out of the 18 articles selected for quality assessment, seven were randomized controlled trials (RCTs), three were systematic reviews, one was a Mendelian randomization (MR) analysis, one was a post hoc analysis, and six were cross-sectional studies.

To assess the quality of these articles, the following tools were used: (1) for cross-sectional studies and MR analysis, Joanna Briggs Institute's (JBI) quality appraisal questionnaires [19], (2) for systematic reviews, Assessment of Multiple Systematic Reviews (AMSTAR) tool [20], and for RCTs and post hoc analysis, Cochrane risk-of-bias assessment tool [21]. These served as valuable tools in evaluating the methodological rigor, validity, and reliability of each study.

Following the quality assessment, we identified eight research articles that demonstrated a quality score exceeding 70%. These eight articles were considered suitable for inclusion in our systematic review.

**Figure 1:**
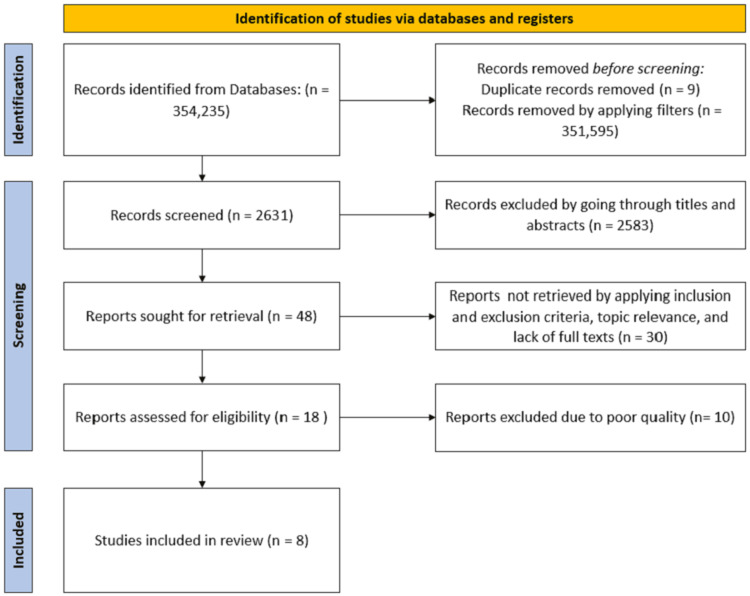
Flow diagram illustrating the article selection process, adhering to the PRISMA guidelines. PRISMA, Preferred Reporting Item for Systematic Review and Meta-Analyses; n, number of articles.

The selection process and the flow of article screening are visually presented in Figure 1, providing a clear overview of the article selection procedure.

Characteristics of the Included Studies

The final review comprised a total of eight selected studies, including four cross-sectional studies, three RCTs, and one MR analysis. Among these studies, two were conducted in Austria [1,22] while the others were conducted in Iran [23], China [24], Malaysia [9], Korea [25], Dutch [26], and the USA [27]. The study participants were adult males aged 18 years and above, and sample sizes in these studies ranged from 62 to 4254. The inclusion criteria encompassed studies published between January 2013 and April 2023. Most studies utilized morning blood samples to assess serum testosterone and vitamin D levels. In the RCTs, testosterone levels were measured after 12 weeks of vitamin D supplementation [1,22]. One study employed a range of assessment tools, including an Individual and Fertility Information Questionnaire, a semi-quantitative Food-Frequency Questionnaire (FFQ), a Sun Exposure Checklist, a Spermogram Record Sheet, a Drug Side-Effects Record Sheet, and a Vitamin D3 Supplemental Checklist. Table 1 shows the characteristics and results of the eight research articles in our systematic review.

**Table 1:** The features and outcomes of research articles included in the systematic review LH, luteinizing hormone; FSH, follicle-stimulating hormone; TT, total testosterone; FAI, free androgen index; FT, free testosterone.

Author	Study title	Study type	Country of study	Age of the study population	Sample size	Study results
Lerchbaum, E et al. [1]	Effects of vitamin D supplementation on androgens in men with low testosterone levels: a randomized controlled trial	RCT	Austria	≥18	100	Participants received either a placebo or 20,000 IU of oral vitamin D weekly for 12 weeks. No significant differences were observed in testosterone levels or other endocrine parameters between the intervention and placebo groups.
Amini L et al. [23]	Evaluation of the effect of vitamin D3 supplementation on quantitative and qualitative parameters of spermograms and hormones in infertile men: A randomized controlled trial	RCT	Iran	35-39	62	Participants received either a placebo or 50,000 IU of Vitamin D3 once a week for eight weeks. No significant differences were observed in spermogram parameters, LH, FSH, TT, and FAI levels between the two groups. However, the intervention group had decreased SHBG levels (p = 0.01), while the placebo group showed increased FT levels (p = 0.03).
Chi Chen et al. [24]	Causal link between vitamin D and total testosterone in men: a Mendelian randomization analysis	MRA	China	≥18 years old	4,254	The study revealed that lower vitamin D levels (25(OH)D) were linked to reduced total testosterone (T) levels. Genetic risk for lower vitamin D (VD_GRS) was also associated with decreased 25(OH)D and total T levels. Mendelian randomization analysis has indicated a potential causal relationship, wherein elevated 25(OH)D levels are associated with a 0.12 increase in total T. Adequate vitamin D is essential for proper testosterone regulation.
Kok-Yong Chin et al. [9]	Vitamin D is significantly associated with total testosterone and sex hormone-binding globulin in Malaysian men	Cross-sectional	Malaysia	20 or above	382	Individuals with vitamin D deficiency (25-hydroxyvitamin D levels below 50 nmol/L) exhibited notably reduced levels of total testosterone and sex hormone-binding globulin (SHBG) in comparison to those with vitamin D insufficiency and optimal levels (statistically significant with p <0.05). However, these differences became statistically nonsignificant after adjusting for body mass index (BMI) (p > 0.05).
Lerchbaum E et.al. [22]	Vitamin D and testosterone in healthy men: a randomized controlled trial	RCT	Austria	≥18	98	In healthy men with normal 25-hydroxyvitamin D and total testosterone levels, vitamin D treatment did not significantly affect total testosterone. However, it had a significant impact on insulin sensitivity (QUICKI) and showed a trend toward decreasing the Matsuda index
Sim MY et al. [25]	Seasonal variations and correlations between vitamin D and total testosterone levels	Cross-sectional	Korea	25-86	1,559	There was no significant association between serum 25-hydroxyvitamin D (25(OH)D) and total testosterone levels in Korean men. However, both 25(OH)D and total testosterone levels showed significant seasonal variations that remained significant even after adjusting for confounding variables.
Wentz LM et al. [27]	Vitamin D correlation with testosterone concentration in male US soldiers and veterans	Cross-sectional	USA	>25	796	The average serum vitamin D level was 29.2 ± 11.1 ng/mL, with 55.7% having deficient or insufficient levels. Lower vitamin D levels in the lowest quintile were associated with lower testosterone levels, younger age, and higher BMI. However, when accounting for BMI, age, and timing of testosterone measurement, the significance of vitamin D in predicting testosterone levels disappeared.
Rafiq R et al. [26]	Associations of vitamin D status and vitamin D-related polymorphisms with sex hormones in older men	Cross-sectional	Dutch	65-89 years	643	Lower serum 25-hydroxyvitamin D (25(OH)D) levels were associated with reduced total and bioavailable testosterone. Men with 25(OH)D levels below 25 nmol/L, 25-50 nmol/L, and 50-75 nmol/L had lower total testosterone compared to those above 75 nmol/L after accounting for confounding factors. The association between 25(OH)D and bioavailable testosterone was significant only for men with levels below 25 nmol/L. No significant relationships were found with SHBG, estradiol, or gonadotropin levels. Hypogonadism was not linked to lower 25(OH)D levels. Gene polymorphisms related to vitamin D did not significantly affect hormone levels or alter associations between 25(OH)D and sex hormones or gonadotropins.

Discussion

Vitamin D deficiency is a global health concern, affecting a substantial portion of the population with insufficient levels of this vital nutrient [28-31]. Research indicates that approximately one billion individuals exhibit insufficient levels of vitamin D, a concern that spans across various ethnicities and age demographics [28,32]. Moreover, the prevalence of contemporary lifestyles characterized by limited sunlight exposure and dietary diversity has significantly contributed to the pervasive occurrence of insufficient vitamin D levels [33]. In recent years, there has been a growing interest in investigating the potential influence of vitamin D on androgens, particularly testosterone levels, in men [34]. This systematic review brings together a collection of studies that aim to enhance our understanding of the relationship between vitamin D and androgens, shedding light on the implications of vitamin D deficiency for male reproductive health and overall well-being. Through a comprehensive analysis of these studies, we will explore the findings and insights obtained, providing valuable insights into the effects of vitamin D on androgens and testosterone levels. In the subsequent sections, we will delve into specific aspects of this relationship to better understand the impact of vitamin D deficiency on male health.

Association Between Vitamin D and Testosterone Levels

The presence of VDR in the male reproductive system suggests that vitamin D may play a role in the synthesis of male reproductive hormones [5]. Understanding the association between vitamin D and testosterone levels is important for comprehending male reproductive health. Several studies have investigated this relationship, providing valuable insights into the complex interplay between vitamin D and testosterone.

Chen et al. conducted an MR analysis on a cohort of 4254 Chinese men to explore the potential causal relationship between vitamin D deficiency and testosterone levels [24]. Their analysis revealed that a genetic decrease in 25-hydroxyvitamin D (25(OH)D) was associated with lower testosterone levels, suggesting a possible causal role of vitamin D in increasing testosterone levels. However, it is worth mentioning the study conducted by Chen et al. They constructed their vitamin D genetic risk score (VD_GRS) based on common genetic variants associated with vitamin D levels, which may not capture the full spectrum of genetic variations affecting 25(OH)D levels. Additionally, only a small percentage (2.7%) of the study subjects in Chen et al.'s study exhibited severe vitamin D deficiency, limiting the ability to establish a direct correlation between severe vitamin D deficiency and testosterone deficiency [24].

In contrast, Rafiq R et al. conducted a cross-sectional study on 643 Dutch men, which revealed a positive correlation between vitamin D (serum 25(OH)D) levels and both total and bioavailable testosterone levels. Men with lower serum 25(OH)D levels, particularly those below 25 nmol/L, tended to have lower total and bioavailable testosterone levels compared to men with higher serum 25(OH)D levels. This relationship remained statistically significant even after accounting for potential confounding variables. However, it is important to note that the observed differences in testosterone levels were relatively small (ranging from -2.1 to -0.8 nmol/L), and the clinical significance of these differences may vary. Additionally, the association between serum 25(OH)D and bioavailable testosterone was only significant for men with very low vitamin D levels (<25 nmol/L) [26].

Interestingly, serum 25(OH)D levels were not found to be related to sex hormone-binding globulin (SHBG), estradiol, or gonadotropin levels, suggesting that vitamin D status may have a specific influence on testosterone levels rather than affecting the overall hormonal profile [26]. In contrast to the findings of Chen et al., the study found no association between vitamin D-related gene polymorphism and hormonal levels. Furthermore, the study, which had a relatively smaller sample size compared to Chen et al., found that the vitamin D-related gene polymorphisms did not modify the relationships between serum 25(OH)D levels and sex hormones or gonadotropins. This indicates that these genetic variations do not interact with vitamin D to alter the associations between vitamin D status and hormone levels. Although the smaller sample size of this study may limit its statistical power and generalizability, its findings provide additional insights into the specific influence of vitamin D on testosterone levels. Further research with larger sample sizes would be beneficial to corroborate these findings and enhance our understanding of the complex relationship between vitamin D and hormone regulation.

In contrast to the findings of Chen et al., Sim MY et al. conducted a cross-sectional study on 1559 Korean men and found no significant association between 25(OH)D and testosterone levels. However, they did observe significant seasonal variations in serum 25(OH)D and total testosterone (TT) levels [25].

Another cross-sectional study conducted by Chin Kok Yong et al. on 382 Malaysian men provided further insights. This study found an association between 25(OH)D levels and TT as well as SHBG. Notably, the association was dependent on body mass index (BMI), suggesting that BMI may modify the relationship between vitamin D and testosterone levels [9]. These findings align with the results of Chen et al., supporting the potential role of vitamin D in testosterone regulation.

Furthermore, a cross-sectional study conducted by LM Wentz et al. in 796 US male soldiers and veterans revealed a high prevalence of inadequate vitamin D levels among male military personnel. Men with the lowest vitamin D levels exhibited notably lower concentrations of testosterone compared to those with the highest vitamin D levels. Additionally, a weak positive correlation between vitamin D levels and TT was observed, particularly among men with deficient or insufficient vitamin D status [27]. These findings imply that maintaining sufficient vitamin D levels could potentially play a role in supporting testosterone levels.

Nevertheless, after considering factors such as age, BMI, and the timing of testosterone measurement, the relationship between vitamin D and testosterone appeared to weaken, suggesting that these variables may have an impact on the association between them. Age and BMI, for example, can independently affect testosterone production and metabolism. Additionally, the timing of testosterone measurement can introduce variability as testosterone levels fluctuate throughout the day.

While the studies provide evidence supporting the role of vitamin D in testosterone regulation, they also highlight the importance of considering other factors that can influence this relationship. In particular, the findings support the Chin et al. study, which suggests that BMI may modify the relationship between vitamin D and testosterone levels. This implies that individuals with different BMI categories may exhibit varying responses to vitamin D in terms of testosterone regulation.

Overall, the studies by Chen et al., Rafiq R et al., Sim MY et al., Chin et al., and LM Wentz et al. provide valuable and insightful information regarding the connection between vitamin D and testosterone levels. However, further research is needed to fully understand the complex relationship and the potential influence of genetic variations, seasonal variations, and other factors such as BMI, age, and timing of testosterone measurements. These findings contribute to the existing body of knowledge and emphasize the importance of comprehensive investigation to enhance our understanding of the interplay between vitamin D and testosterone in the context of male reproductive health.

Effects of Vitamin D Supplementation on Testosterone Levels

The influence of vitamin D on testosterone levels has been extensively investigated in various studies. In this section, we delve into the effects of vitamin D supplementation on testosterone, shedding light on the potential therapeutic role of vitamin D in modulating hormonal balance.

Lerchbaum Elisabeth et al. conducted an RCT involving 94 men with low testosterone levels (TT levels < 10.4 nmol/L) in Austria to explore the causal relationship between vitamin D and testosterone levels. The study did not find a significant treatment effect of vitamin D supplementation on testosterone levels compared to the placebo group [1]. However, it is important to consider that the relatively short duration of the study (12 weeks) may have limited the ability to detect significant changes in testosterone levels over a longer-term supplementation period. Furthermore, the use of weekly vitamin D administration in the study design could have influenced the observed results.

Notably, the study revealed a significant increase in SHBG levels in the placebo group among men with lower vitamin D status, while SHBG levels remained unchanged in the vitamin D group [1]. This finding suggests that vitamin D supplementation may have a stabilizing effect on SHBG levels in individuals with lower vitamin D status, indicating a potential role of vitamin D in regulating the availability of sex hormones in the body.

Lerchbaum Elisabeth et al. also conducted another RCT on 98 middle-aged healthy men with TT levels ≥10.4 nmol/L in Austria. The findings of this study align with the previous RCTs conducted on men with low testosterone levels (TT < 10.4 nmol/L). The study showed that vitamin D treatment did not have a significant impact on TT levels [22]. However, it is important to note that these findings are specific to middle-aged healthy men with normal baseline testosterone levels. Therefore, the conclusions cannot be generalized to other populations, such as individuals with testosterone deficiencies. Additionally, the short duration of vitamin D supplementation and the weekly dosing regimen employed in the study may have influenced the results.

In a similar vein, Amini Leila et al. conducted an RCT examining the effects of vitamin D3 supplementation on 62 infertile men with impaired spermatogonial tests. The results indicated no significant differences between the vitamin D3 supplementation group and the placebo group in terms of spermogram parameters or serum levels of LH (luteinizing hormone), FSH (follicle-stimulating hormone), TT, FT (free testosterone), SHBG, and FAI (free androgen index) [23]. These findings suggest that vitamin D3 supplementation did not have a significant impact on these specific measures. However, notable changes were observed within each group. In the intervention group, there was a significant decrease in SHBG levels after the intervention, potentially indicating an increase in the availability of FT, the biologically active form of testosterone. Interestingly, in the placebo group, there was a significant increase in FT levels [23], which contrasts with the findings of Lerchbaum Elisabeth et al., where the placebo group showed a significant increase in SHBG levels [1]. However, it is important to note that the treatment group received weekly doses of 50,000 IU of vitamin D3 for eight weeks, followed by a maintenance dose of vitamin D3 (50,000 IU) once a month for the remaining four weeks. This relatively short duration of supplementation may have influenced the observed results.

In conclusion, the studies examining the effects of vitamin D supplementation on testosterone levels have provided valuable insights, but the findings remain inconclusive. While there is no significant impact on TT levels, notable changes in SHBG and FT levels have been observed within the intervention and placebo groups. These findings suggest potential influences of vitamin D supplementation on hormonal balance, particularly in individuals with impaired spermatogonial tests. However, variations in study duration, dosing regimens, and participant characteristics emphasize the need for further research to elucidate the true effects of vitamin D supplementation on testosterone and its related parameters.

Study Limitations

Several limitations should be acknowledged in relation to the reviewed studies. First, there was considerable heterogeneity in the design, sample size, and duration of vitamin D supplementation among the included studies, which may limit the generalizability of the findings and hinder the ability to draw definitive conclusions. The majority of studies were either cross-sectional or RCTs with relatively short durations, potentially overlooking the long-term effects of vitamin D supplementation on testosterone levels. Moreover, the focus of the studies was predominantly on specific populations, such as men with low testosterone levels or those with impaired spermatogonial tests, which may restrict the generalizability of the findings to broader populations. Additionally, variations in the measurement and assessment of testosterone levels across the studies introduce the possibility of variability and could affect the comparability of results. These limitations underscore the need for well-designed, long-term RCTs with diverse populations to establish a more comprehensive understanding of the relationship between vitamin D and testosterone levels.

## Conclusions

In summary, the reviewed studies have provided valuable insights into the complex relationship between vitamin D and androgens, specifically testosterone levels, in men. The evidence regarding this association has yielded conflicting findings, with some studies showing a positive correlation while others failing to find a significant link. Similarly, the effects of vitamin D supplementation on testosterone levels remain inconclusive, with limited evidence of notable changes in TT but potential influences on SHBG and FT levels. It is important to acknowledge the limitations of the included studies, such as variations in study design, sample size, duration of supplementation, and participant characteristics. These limitations, along with the heterogeneity of the findings, underscore the necessity for well-designed, long-term RCTs encompassing diverse populations. Only through such comprehensive investigations can we hope to unravel the complexities of the relationship between vitamin D and testosterone in men, thus advancing our understanding of this pivotal aspect of male health and physiology.

## Appendices

**Table 2:** Summary of the JBI Critical Appraisal Tool for Cross-Sectional studies. Quality check was done as per the JBI Critical Appraisal Scale (Yes, No, Unclear, N/A). JBI, Joanna Briggs Institute.

Selection	Kok-Yong Chin et al. [9]	Chi Chen et al. [24]	Sim MY et al. [25]	Rafiq R et al. [26]	LM Wentz et al. [27]	Ningjian Wang et al. [35]	Anna Książek et al. [36]
Were the criteria for inclusion in the sample clearly defined?	No	Yes	Yes	Yes	Yes	No	No
Were the study subjects and the setting described in detail?	Yes	Yes	Yes	Yes	Yes	Yes	Yes
Was the exposure measured in a valid and reliable way?	Yes	Yes	Yes	Yes	Yes	Yes	Unclear
Were objective, standard criteria used for measurement of the condition?	Yes	Yes	Yes	Yes	Yes	Yes	Yes
Were confounding factors identified?	Yes	Unclear	Unclear	Yes	No	Yes	Unclear
Were strategies to deal with confounding factors stated?	Yes	Unclear	Unclear	Unclear	No	Unclear	Unclear
Were the outcomes measured in a valid and reliable way?	Yes	Yes	Yes	Yes	Yes	Yes	Yes
Was appropriate statistical analysis used?	Yes	Yes	Yes	Yes	Yes	No	Yes
Total	7/8	6/8	6/8	7/8	6/8	5/8	4/8
Quality %	88%	75%	75%	88%	75%	63%	50%
